# Dysgerminoma in a case of 46, XY pure gonadal dysgenesis (swyer syndrome): a case report

**DOI:** 10.1186/1746-1596-6-84

**Published:** 2011-09-19

**Authors:** Yang Han, Yan Wang, Qingchang Li, Shundong Dai, Anguang He, Enhua Wang

**Affiliations:** 1 Department of Pathology, College of Basic Medical Sciences and First Affiliated Hospital of China Medical University, Shenyang, 110001 China

**Keywords:** dysgerminoma, Swyer syndrome, 46, XY pure gonadal dysgenesis

## Abstract

Simple 46, XY gonadal dysgenesis syndrome, also called Swyer syndrome, is known as pure gonadal dysgenesis. Individuals with the syndrome are characterized by 46, XY karyotype and phenotypically female with female genital appearance, normal Müllerian structures and absent testicular tissue. The condition usually first becomes apparent in adolescence with delayed puberty and primary amenorrhea due to the gonads have no hormonal or reproductive potential. Herein, we report a case of dysgerminoma diagnosed in a dysgenetic gonad of a 21-year-old patient with Swyer syndrome.

## Background

Doctor Swyer described two women whom had a 46, XY karyotype, tall stature, primary amenorrhea, female external genitalia, normal vagina (albeit hypoestrogenised) and cervix in 1955 [1]. Individuals with Swyer syndrome are phenotypically female with unambiguously female genital appearance from birth and normal Müllerian structures. The patients usually first become apparent in adolescence with delayed puberty and primary amenorrhea due to the fact that the gonads have no hormonal or reproductive potential. Here, we report a case of dysgerminoma diagnosed in a dysgenetic gonad of a 21-year-old patient with Swyer syndrome.

## Clinical history

A 21 years old patient with a history of primary amenorrhea, 46, XY karyotype, was admitted to the hospital complainging of a low abdominal mass. The patient is 164 cm tall. She has breast development (tanner IV). The pubic hair distribution is adult in type but decrease in total quantity. Her menstruation had not started until she took estradiol valerate and medroxyprogesterone for 15 months, which was prescribed by doctor in her 17 years old. In 19 years old, menstruation started, 6 days at intervals of 23-40 days, blood is ruby-red, sometimes blood clot. However, menstruation disappeared at the age of 20 because she stopped the pills. Chromosome analysis with fluorescence in situ hybridization revealed 46, XY. Color doppler imaging (CDI) showed the uterus was 3.22 × 5.19 × 2.80 cm, thickness of the endometrium was 0.19 cm thick. There was a heterogenetic mixed echo (5.50 × 3.77 cm) behind the cervix. Laboratory investigations showed CA125 was 11.53 U/ml (normal 0.00 ~ 35.00 U/ml), testosterone free (F-TEST) was 34.5 pmol/L (normal 0.77 ~ 33.03 pmol/L), FSH was 56.70 mIU/ml, LH was 21.50 mIU/ml, sex hormone binding globulin (SHBG) was 16.50 nmol/L (normal 18.0 ~ 114.0 nmol/L) and carcino-embryonic antigen (CEA) was 0.55 ng/ml (normal 0 ~ 4.3 ng/ml). Exploratory laparotomy was performed. Surgical findings: The uterus was small (3.0 × 5.0 × 2.0 cm). A 5.0 × 5.0 × 4.0 cm firm mass involved the right gonad with a smooth surface and good mobility. The left gonad was aplasia, like streak and without follicle. The bilateral fallopian tubes were slender. The serosa of the liver, the spleen and the stomach were smooth. There was no obvious nodule in the omentum. The result of the frozen section is malignant tumor of the ovary. Based on the agreement with the patient and her family bilateral salpingoopherectomy and hysterectomy (BSOH) and lymph node dissection were performed. Due to tumor was classified as stageIA, adjuvant therapy was not commented. Written consent for publication was obtained from the patient.

## Pathology

### Gross

**Figure 1:**
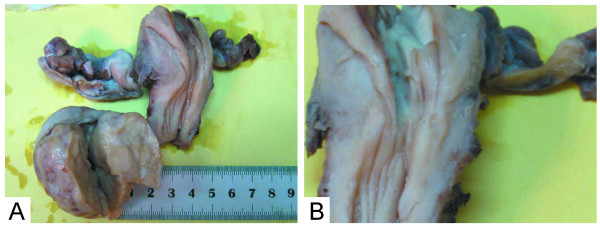
**The right gonad was a 5.0 × 5.0 × 4.0 cm firm mass, encapsulated, with gritty gray-yellow cut surface**. The cut surface of the tumor was solid, fleshy, lobulated and gray (A). The double fallopian tubes were slender. The uterus was small (3.0 × 5.0 × 2. 0 cm) and the endometrium was smooth. The left gonad was 2.0 × 0.6 × 0.6 cm and appeared as streak gonad (B).

A 5.0 × 5.0 × 4.0 cm firm mass involved the right gonad, while the left gonad appeared as streak gonad measuring 3.0 × 0.6 × 0.6 cm. The tumor was encapsulated with a smooth surface. The cut surface of the tumor was solid, fleshy, lobulated and gray-yellow (Figure 1A). The bilateral fallopian tubes were slender. The uterus was small (3.0 × 5.0 × 2.0 cm) and the endometrium was smooth. The left gonad was 2.0 × 0.6 × 0.6 cm and appeared as streak gonad (Figure 1B).

### Histology and Immunohistochemistry

The tumor and the other abscised tissues were fixed in 10% formalin and embedded in paraffin. Several 4-μm sections were cut from each paraffin block, and one was stained with HE (hematoxylin and eosin), the others were stained with IHC (immunohistochemistry). Immunohistochemical staining was performed using the streptavidin-peroxidase system (Ultrasensitive; MaiXin Inc., Fuzhou, China) according to the manufacturer's instruction. Commercially available prediluted monoclonal antibodies against the following antigens were employed: CK(cell keratin), CD30, PLAP (placental alkaline phosphatase), CD117 (all Thermo Fisher Scientific Inc., Fremont, CA, USA).

## Results

**Figure 2:**
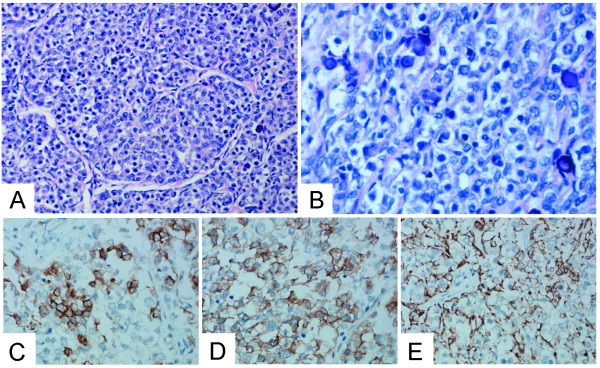
**The tumor cells grouped themselves in well-defined nests separated by fibrous strands infiltrated by lymphocytes**. The tumor cell membrane was prominent. The uniform, rounded tumor cells have clear to eosinophilic cytoplasm, and a central, large, rounded or flattened nucleus that contains one or a few prominent nucleoli. Calcification was seen(A, HE × 200 B, HE × 400). Immunohistochemically, the tumor cells were positive for PLAP(C, × 400), CD117(D, × 400), and CK(E, × 400), but negative for CD30.

Microscopically, the tumor in the right dysgentic gonad showed the typical features of dysgerminoma. The tumor cells grouped themselves in well-defined nests separated by fibrous strands infiltrated by lymphocytes. The tumor cell membrane was prominent. The uniform, rounded tumor cells have clear to eosinophilic cytoplasm, and a central, large, rounded or flattened nucleus that contains one or a few prominent nucleoli. Calcification was seen (Figure 2A~B). No metastases were detected. Immunohistochemically, the tumor cells were positive for PLAP, CD117, and CK, but negative for CD30. (Figure 2C~E)

**Figure 3:**
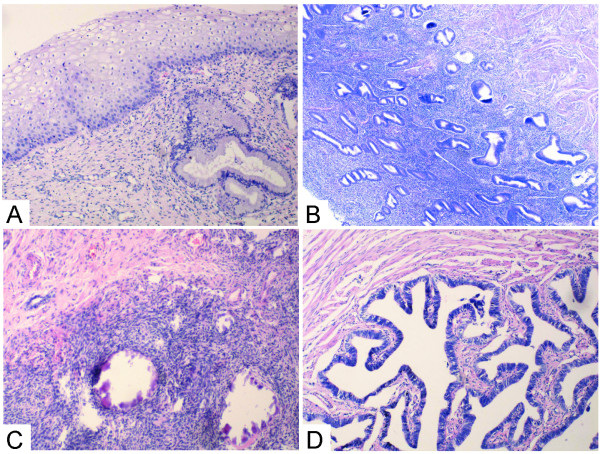
**The squamous cells of the cervix was normal, and lymphocytes infiltrated was seen in the cervix tissue (A HE × 100)**. The endometrium was in early proliferative phrase (B HE × 40). The left streak gonad with ovarian-type stroma, no primordial ovarian follicles and calcification was seen (C HE × 100). The tissue structure of the fallopian tubes were normal (D HE × 200).

The squamous cells of the cervix was normal, and lymphocytes infiltrated was seen in the cervix tissue. (Figure 3A). The endometrium was in early proliferative phrase--thin surface epithelium; straight, short, narrow glands and compact stroma (Figure 3B). The left streak gonad had ovarian-type stroma (fibrous gonad), no primordial ovarian follicles and calcification was seen (Figure 3C). The fallopian tubes were slender and the tissue structure were normal (Figure 3D).

## Discussion

The Swyer syndrome, 46, XY gonadal dysgenesis, belongs to the category of sexual abnormality [2]. The syndrome was complete/pure gonadal dysgenesis. The patients with 46, XY gonadal dysgenesis are diagnosed in early adolescence with delayed pubertal development. The patients' mesonephric ducts (Wolffian ducts) are in atrophy, paramesonephric ducts (Müllerian ducts) develops to uterus, fallopian tubes and part of the vagina as a result of lacking testosterone and inhibitor of Müllerian ducts. As expected they show elevated gonadotropins, normal female levels of androgens, low levels of estrogens, female external genitalia, uterus and fallopian tubes. Minimal breast enlargement reflects peripheral aromatization of androgens. Both gonads display fibrous tissue that vaguely resembles ovarian stroma but no follicles. The etiology of 46, XY pure gonad dysgenesis is thought to be a shot arm Y chromosome deletion involving SRY (putative testicular-determining factor gene), a mutation in other genes that leads to inhibition of SRY function or mutation of SRY function [3].

Swyer syndrome should be differentiated from the following two female phenotype (karyotype XY) syndrome. One is the familial syndrome of testicular feminization, the most common type of male pseudohermaphroditism. It occurs in individuals with a normal male chromosome constitution with an end-organ defect (androgen insensibility). It characterized by the presence of several well-developed female secondary sex characteristics. Individuals are found to have a vagina (not a true vagina, but like a small "cupule"), bilateral cryptorchid testes but no uterus [4,5]. The other is true hermaphrodites, which may have ovotestes containing both ova and immature seminiferous tubules or other combinations of ovary and testis [6,7].

In patients with gonadal dysgenesis, either "pure"(with a 46,XX or 46, XY karyotype) or associated with the somatic features of Turner's syndrome (with a 45,XO karyotype), both gonads are represented by a streak of fibrous tissue that vaguely resembles ovarian stroma [8,9]. Previously thought these patients do not seem to have an increased incidence of gonadal tumors [10]. But modern studies show patients with 46, XY pure gonadal dysgenesis are at a higher risk of developing gonadoblastoma and dysgerminoma, and may occur even in young ages. The incidence of Swyer syndrome is 1:100 000 [11,12]. A bilateral gonadectomy should be done especially by laparoscopy when a Swyer syndrome is discovered in order to avoid the risk of malignant transformation. There is possibility of pregnancy by oocyte donation in some countries if the uterus was not removed for a malignant etiology.

In this report, we provide a case of dysgerminoma diagnosed in a dysgenetic gonad of a 21-year-old patient with Swyer syndrome, who presented with primary amenorrhea and infertility of five years duration. Karyotype was consistent with 46, XY (pure). In our case, testosterone free was 34.5 pmol/L, sex hormone binding globulin was 16.50 nmol/L, and menstrual function was associated with estradiol valerate and medroxyprogesterone prescribed by doctor. Estrogen and progestin sequential therapy supports female secondary sex development in patients with gonadal dysgenesis. The patient did not have a bilateral gonadectomy after knowing she has a 46, XY caryotype because she wanted to be pregnant and did not believe she had the risk of suffering from malignent of ovary. It is necessary to take the familial screening of Swyer syndrome cases. As a malignant germ cell tumor of the ovary, dysgerminoma can be found either in a pure form or mixed with other germinal elements. Therefore in premenarchal patients with a pelvic mass, the karyotype should be determined.

Differential diagnosis for dysgerminoma include diffuse large B cell lymphoma, poorly differentiated carcinoma, embryonal carcinoma and gonadoblastoma. Coexistence of dysgerminoma and gonadoblastoma is seen in about 50% of cases [13]. Histologic appearance of gonadoblastoma may be altered by hyalinization, calcification, and/or overgrowth of a malignant germ cell element predominantly dysgerminoma. Maleki et al. reported a case of dysgerminoma and gonadoblastoma in a dysgenetic gonad on a touch preparation that described cytomorphologic findings of both neoplasms [14].

About 65% of dysgerminomas are stage I at diagnosis. About 85 ~ 90% of stage I tumors are confined to one ovary; 10 ~ 15% are bilateral. Dysgerminoma is the only germ cell malignancy that has this significant rate of bilaterality, other germ cell tumors being rarely bilateral. The treatment of patient with early disgerminoma is resection of the primary lesion and proper surgical staging. Chemotherapy and/or radiotherapy are administered to patients with metastases. In patients whose contralateral ovary has been preserved, some disease can develop in 5 ~ 10% of the retained gonads over the next 2 years [15].

## Competing interests

The authors declare that they have no competing interests.

## Authors' contributions

YH analyzed the data and wrote the manuscript as a major contributor. YW, QL and SD contributed to management of the patient. AH and EW carried out the histopathological evaluation and helped to write manuscript. All authors have read and approved the final manuscript.
